# Post hoc analysis of reactogenicity trends between dose 1 and dose 2 of the adjuvanted recombinant zoster vaccine in two parallel randomized trials

**DOI:** 10.1080/21645515.2020.1741312

**Published:** 2020-04-29

**Authors:** Romulo Colindres, Valentine Wascotte, Alain Brecx, Christopher Clarke, Caroline Hervé, Joon Hyung Kim, Myron J. Levin, Lidia Oostvogels, Toufik Zahaf, Anne Schuind, Anthony L. Cunningham

**Affiliations:** a GSK, Rockville, MD, USA; b independent Biotech and Vaccines Consultant; c GSK, Wavre, Belgium; d GSK, Rixensart, Belgium; e Seqirus, Victoria, Australia; f Departments of Pediatrics and Medicine, University of Colorado Anschutz Medical Campus, Aurora, CO, USA; g CureVac AG, Tübingen, Germany; h The Westmead Institute for Medical Research, University of Sydney, Sydney, Australia

**Keywords:** Recombinant herpes zoster vaccine, reactogenicity, doses, healthcare professionals, adverse events

## Abstract

In two large clinical trials (ZOE-50 [NCT01165177] and ZOE-70 [NCT01165229]), two doses of the adjuvanted recombinant zoster vaccine (RZV) demonstrated >90% efficacy against herpes zoster in adults ≥50 years of age. Solicited adverse events (AEs) were collected for 7 days post-each dose in a study sub-cohort. The incidence of reported solicited AEs was higher for RZV compared to placebo recipients. Since reactogenicity may contribute to a person’s willingness to be vaccinated, knowing about expected reactogenicity might help keep high compliance with the second dose. This post hoc analysis assessed the intensity of solicited AEs post-dose 2 reported to the same event’s intensity post-dose 1. Intensity was graded from 0 to 3, grade 3 indicating the highest severity. Of the vaccinees who did not experience a specific AE post-dose 1, 72.6–91.7% did not experience the same event after dose 2. Although the frequency of grade 3 AEs post-dose 2 was the highest in participants reporting the same AEs at grade 3 post-dose 1, 65.8–89.3% of vaccinees with grade 3 specific AEs post-dose 1 reported the same AEs at lower intensity post-dose 2. These data can help inform health-care professionals about the frequency and intensity of AEs post-dose 2 with respect to post-dose 1.

Herpes zoster (HZ) causes a significant burden of disease in adults over 50 years of age (YOA).^1^ Two doses of the adjuvanted recombinant zoster vaccine (RZV, *Shingrix*, GSK) demonstrated an acceptable safety profile and high efficacy against HZ and postherpetic neuralgia (PHN).^2–4^ RZV is licensed and recommended for the prevention of HZ and PHN in adults ≥50 YOA in several countries worldwide.

RZV consists of varicella-zoster virus glycoprotein E (gE) and the liposome-based AS01_B_ Adjuvant System (containing 50 μg of 3-O-desacyl-4ʹ-monophosphoryl lipid A and 50 μg of *Quillaja saponaria* Molina, fraction 21 [licensed by GSK from Antigenics LLC, a wholly owned subsidiary of Agenus Inc., a Delaware, USA corporation]). The resulting formulation enhances cell-mediated immune responses and overcomes immunosenescence,^5^ leading to a vaccine with high efficacy in older adults.^2,3^

It has previously been observed that adjuvanted vaccines may be associated with increased reactogenicity.^6–10^ Indeed, occurrence of solicited adverse events (AEs) during the 7-day post-vaccination period was higher in RZV compared to placebo recipients in the two pivotal phase III trials conducted in adults ≥50 YOA (ZOE-50^11^) and ≥70 YOA (ZOE-70^3^): 85.2% (RZV) compared to 34.2% (placebo) in ZOE-50 and 79.0% (RZV) compared to 29.5% (placebo) in ZOE-70. The most common solicited adverse events were pain at the injection site, myalgia, fatigue, and headache.^3,11,12,13^ While the majority of events reported during the 7-day post-vaccination period were mild to moderate, with median durations between 1 and 3 days, 16.4% (ZOE-50) and 11.9% (ZOE-70) of events were reported at grade 3 intensity.^3,11^

In clinical settings, the high efficacy of RZV was demonstrated following a 2-dose vaccination schedule.^2,3^ Therefore, 2 dose-compliance is recommended to ensure optimal protection. As AEs experienced after the first dose could impact adherence to the subsequent dose, second-dose compliance may be improved if vaccine recipients and physicians are well informed on expected reactogenicity during the course of vaccination.^7^ Herein, we present a post hoc analysis assessing the intensity of a specific injection site and general AE after RZV dose 2 with respect to the intensity of the same event after RZV dose 1.

ZOE-50 and ZOE-70 were phase III, randomized, observer-blind, controlled trials, conducted in parallel in 18 countries in Asia, Australia, Europe, Latin America, and North America in adults ≥50 YOA (NCT01165177) and ≥70 YOA (NCT01165229). Protocol summaries are available at http://www.gsk-clinicalstudyregister.com (studies 110390 and 113077). Anonymized individual participant data and study documents can be requested for further research at www.clinicalstudydatarequest.com. Participants in both studies were randomized 1:1 to receive 2 doses of either RZV or saline placebo 2 months apart. Inclusion and exclusion criteria have been described previously.^2,3^

The studies had a similar design and were conducted concurrently at the same centers, allowing the pooling of safety data. Hence, the main safety analysis of RZV was performed on the pooled total vaccinated cohort (TVC) from the ZOE-50 and ZOE-70 population, which included all participants with at least one administered dose. Reactogenicity was evaluated in a sub-cohort of participants (TVC reactogenicity) that recorded solicited injection site (pain, redness, and swelling) and general events (fatigue, fever, gastrointestinal symptoms, headache, myalgia, and shivering) on diary cards for 7 days after each vaccination. Intensity of each AE was graded as described in Table 1 and the intensity of each solicited injection site and general event after dose 1 was compared to the intensity of the same event reported after dose 2.

**Table 1.** Grading of solicited adverse events

	Grading			
	0	1	2	3
**Injection site events**				
Pain	None	Mild: Any pain neither interfering with nor preventing normal every day activities	Moderate: Painful when limb was moved and interfered with every day activities.	Severe: Significant pain at rest. Prevented normal every day activities.
Redness (diameter)				
	<20 mm	20–50 mm	>50–100 mm	>100 mm
Swelling (diameter)				
Fatigue				
**General events**				
GI symptoms				
Headache	None	Mild: Event that was easily tolerated	Moderate: Event that interfered with normal activity	Severe: Event that prevented normal activity
Myalgia				
Shivering				
Fever (body temperature)	<37.5°C	37.5–38.0°C	38.1–39.0°C	>39.0°C

GI, gastrointestinal (nausea, vomiting, diarrhea and/or abdominal pain)

The pooled TVC from the ZOE-50 and ZOE-70 studies consisted of 14,645 RZV and 14,660 placebo recipients. Occurrences of unsolicited AEs, serious adverse events (SAEs), and potential immune-mediated diseases (pIMDs) and reasons for exclusions from this pooled cohort have been presented previously.^4^ Of the RZV recipients included in the pooled TVC, 4,969 were included in the reactogenicity sub-cohort (as per protocol).

The overall compliance with the 2-dose schedule was comparably high in RZV and Placebo groups in the pooled TVC. Of the first-dose recipients, 730 (5.0%) participants in the RZV group and 581 (4.0%) in the Placebo group did not receive the second dose. In the RZV and Placebo groups, respectively, 155 (1.1%) and 57 (0.4%) participants did not receive the second dose due to a non-serious AE, while 59 (0.4%) and 61 (0.4%) did not receive the second dose due to SAE/pIMD (Supplementary Table 1).

Of the 432 vaccinees from the reactogenicity sub-cohort who reported any grade 3 injection-site or general events after the first dose, 394 (91.2%) returned to receive their second dose.

Any solicited injection site AEs were recorded after both RZV doses by 4,676 vaccinees (94.1% of the reactogenicity sub-cohort). Pain was the most frequent solicited injection site event after each dose (70.3% [95% confidence interval (CI): 69.0–71.6] after dose 1 and 65.7% [64.4–67.1] after dose 2) (Figure 1). The proportion of RZV recipients reporting any-grade redness and swelling were similar between doses. Of the 4,676 vaccinees, 244 (5.2%) experienced a grade 3 injection site AE after dose 1; 165 (67.6%) of them reported the same event at a lower intensity (≤ grade 2) after dose 2. Of the 1,235 (26.4%) participants who experienced grade 0 injection site AE after dose 1, 881 (71.3%) experienced the event at grade 0 intensity as well after dose 2 (Figure 2). Amongst participants who experienced a certain injection site AE at grade 3 intensity after dose 1, 65.8% to 84.0% of the participants reported the same event at a lower intensity (≤grade 2) after dose 2. Amongst participants who experienced a grade 0 injection site AE after dose 1, the proportion of participants experiencing the same event at grade 0 intensity after dose 2 ranged between 72.6% and 90.4% (Figure 3(a)).

**Figure 1.**
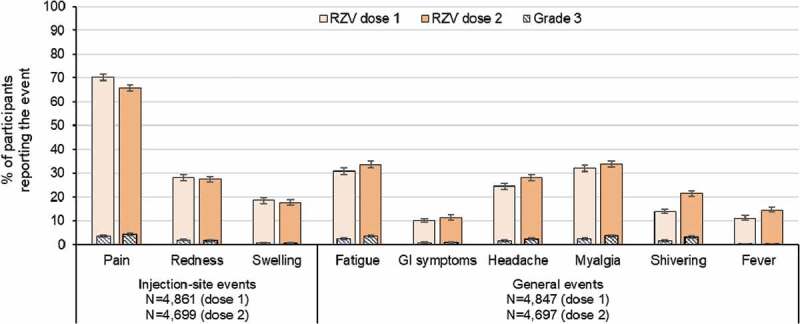
Incidence of solicited injection site and general events reported during the 7-day post-vaccination period following each dose among RZV recipients (TVC reactogenicity)

Footnote: RZV, adjuvanted recombinant zoster vaccine; TVC, total vaccinated cohort; N, number of documented doses; GI, gastrointestinal (nausea, vomiting, diarrhea and/or abdominal pain); fever was defined as body temperature >37.5°C.

**Figure 2.**
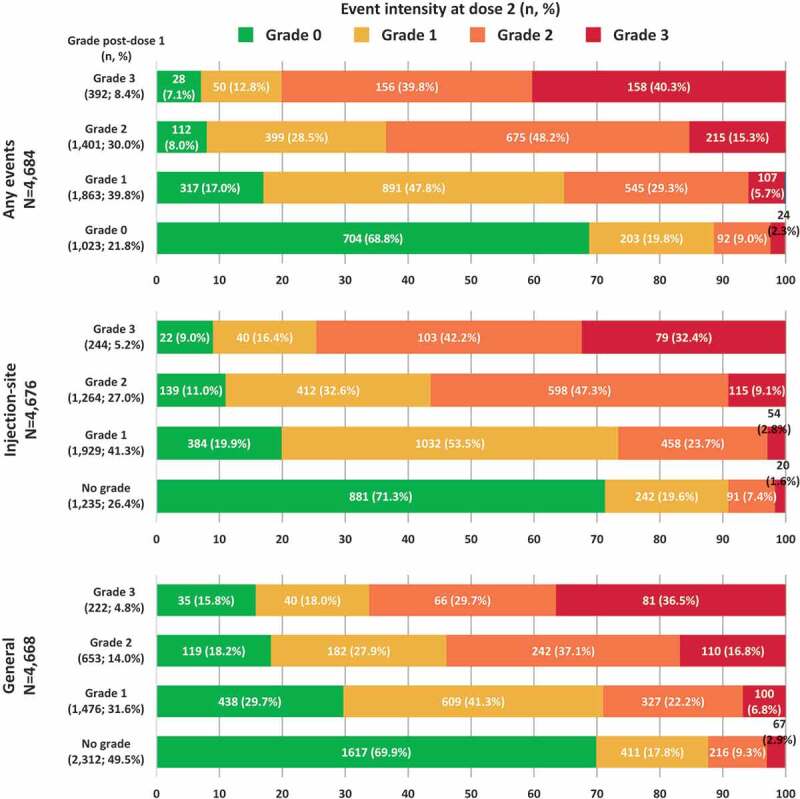
Intensity of any events, solicited injection site events and general events reported after dose 2 stratified by the intensity reported after dose 1 (TVC reactogenicity)

Footnote: TVC, total vaccinated cohort; N, number of participants with both doses administered having the corresponding grade at dose 1; n (%), number (percentage) of RZV vaccinees with events at a specific grade. Note: Injection site events included: pain at the injection site, redness at injection site and swelling at the injection site. General events included any solicited experiences which did not occur at the site of injection of RZV vaccine as: fatigue, gastrointestinal symptoms (nausea, vomiting, diarrhea and/or abdominal pain), headache, myalgia, shivering and fever. There were four injection site events and five general events with missing grading at dose 1 and 6 events (3 for each injection site and general) with missing grading at dose 2.

**Figure 3.**
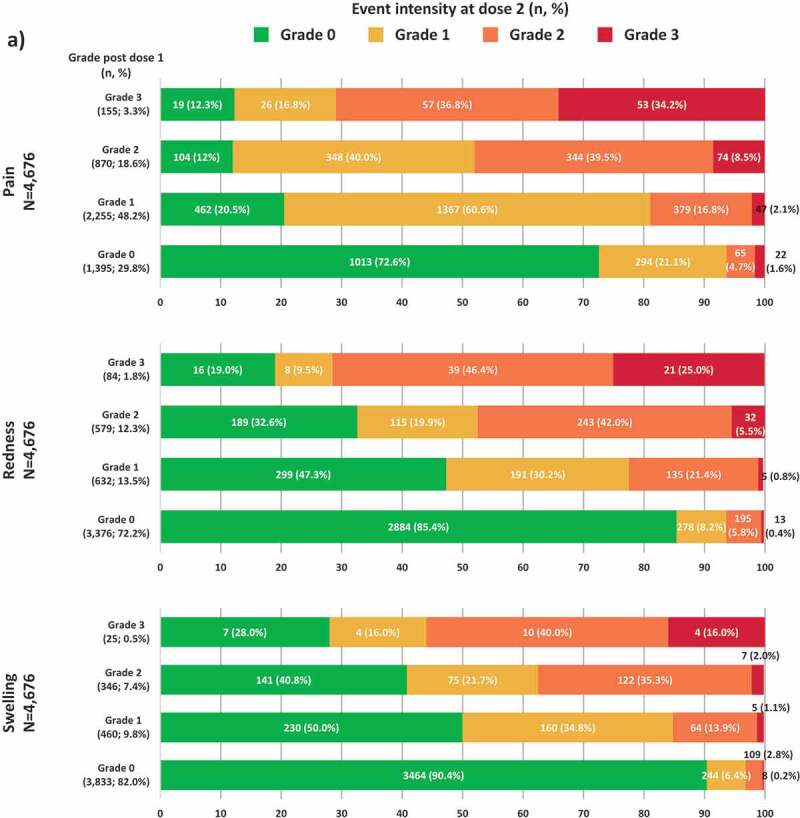
Intensity of solicited injection site (a) and general (b) events reported after dose 2 stratified by the intensity reported after dose 1 (TVC reactogenicity).Footnote: TVC, total vaccinated cohort; N, number of participants with both doses administered having the corresponding grade at dose 1; n (%), number (percentage) of RZV vaccinees with events at a specific grade. Notes: Panel A. Missing grade events: pain (1 event after dose 1 and 2 events after dose 2), redness (5 events after dose 1 and 8 events after dose 2) and swelling (12 events after dose 1 and 10 events after dose 2). Panel B. Missing grade events: GI symptoms (1 event after dose 1 and 1 event after dose 2) and fever (7 events after dose 1 and 6 events after dose 2)

**Figure 3.**
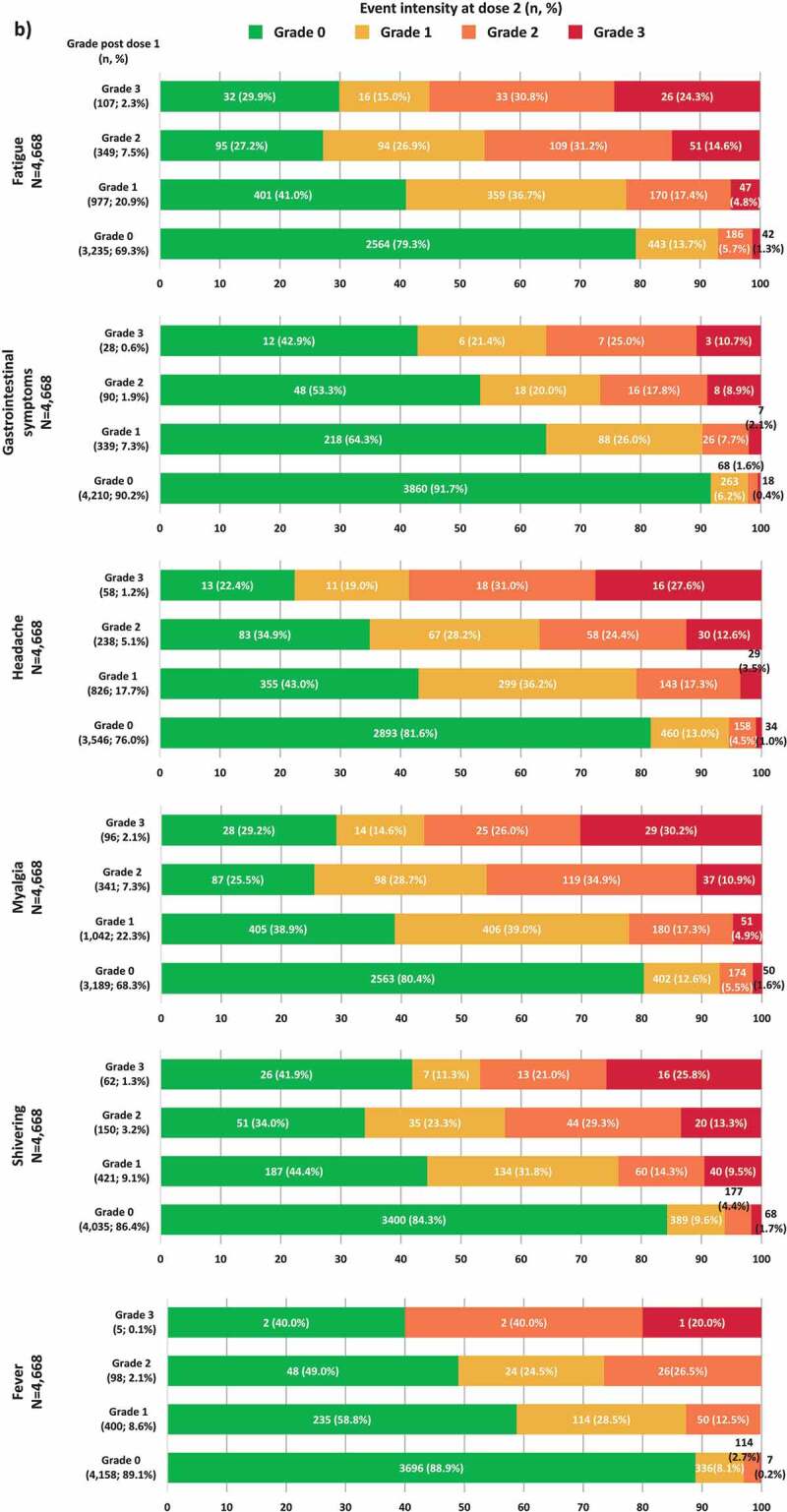
(Continued)

Any solicited general AEs were recorded after both RZV doses by 4,668 vaccinees (93.9% of reactogenicity sub-cohort). Myalgia (32.0% [95%CI: 30.7–33.4] after dose 1 and 33.8% [32.4–35.2] after dose 2), and fatigue (30.9% [29.6–32.2] and 33.6% [32.2–35.0]) were the most frequent solicited general adverse events (Figure 1). While the proportion of RZV recipients reporting myalgia and gastrointestinal symptoms were similar between doses, the proportion of vaccinees reporting fatigue, headache, shivering, and fever was higher after dose 2 than after dose 1. This might be explained by the involvement of immune memory responses induced by the vaccine after the first dose.^14^

Among the 4,668 vaccinees, 222 (4.8%) participants experienced a grade 3 solicited general AE after dose 1; of them, 141 (63.5%) participants reported the same event at a lower intensity (≤grade 2) after dose 2. Of the 2,312 (49.5%) participants who experienced grade 0 solicited general AE after dose 1, 1,617 (69.9%) reported the event at grade 0 intensity as well after dose 2 (Figure 2). Amongst participants who experienced a certain general AE at grade 3 intensity after dose 1 (fatigue, gastrointestinal symptoms, headache, myalgia, shivering, or fever), 69.8% to 89.3% of the participants reported the same event at a lower intensity (≤grade 2) after dose 2. Amongst participants who experienced a grade 0 general AE after dose 1, the proportion of participants experiencing the same event at grade 0 intensity after dose 2 ranged between 79.3% and 91.7% (Figure 3b).

Overall for all solicited injection site and general AEs, vaccinees who did not experience a specific AE after dose 1 generally did not experience the same event after dose 2. Vaccinees reporting grade 3 specific AEs after dose 1 were more likely to report the same event at lower intensity after dose 2 than participants who reported the AE at grade ≤2 post-dose 1. The frequency of grade 3 AE after dose 2 was the highest in participants who reported the same AE at grade 3 intensity after dose 1.

In the pooled TVC, of the 730 (<5% of 14,645 RZV recipients) participants who did not receive the second RZV dose, 214 (29.3%) reported a non-serious AE, SAE, or pIMD. A recent study assessing the impact of the first RZV dose on the daily physical functioning (PF) and quality of life of 401 participants, also demonstrated the acceptable safety profile of the vaccine.^15^ Whereas for participants with grade 1 and grade 2 events the mean Short Form Survey-36 (SF-36) PF score remained stable during days 0, 1, and 2 after dose 1 (84.3, 84.1, and 85.5 [a scale of 0 to 100]), grade 3 reactogenicity was associated with a transient decrease of PF score for 2 days post-vaccination (day 0 – 75.8, day 1 – 65.2, day 2 – 68.0, day 3 – 74.8).^15^ Study results are aligned with the definition of severity of AEs used in our analysis (Table 1).

The post hoc nature of this analysis might be considered as one of its limitations. Moreover, the presented analysis on the reactogenicity post-dose 2 with respect to post-dose 1 is descriptive and thus the results need to be handled with caution. Results are presented as the proportion of vaccinees in sub-groups including widely different numbers of participants; thus, values need to be interpreted with regard to the size of the analyzed sub-group.

Given that clinical trials demonstrated high RZV efficacy for a 2-dose schedule, 2-dose compliance is considered important to prevent shingles in the general older population. While this study was not powered to predict event intensity of the second RZV dose based on the first-dose experience, our data inform healthcare professionals about expected reactogenicity after RZV vaccination and may assist to achieve higher compliance with the second dose.

A plain language summary contextualizing the results and potential clinical research relevance and impact is given in Supplementary Figure 1.

## Supplementary Material

Supplemental Material
