# Supplementary material for: Post hoc analysis of reactogenicity trends between dose 1 and dose 2 of the adjuvanted recombinant zoster vaccine in two parallel randomized trials
Source: Hum Vaccin Immunother. 2020 Apr 29;16(11):2628–33. doi: 10.1080/21645515.2020.1741312 (PMC7733973; doi:10.1080/21645515.2020.1741312)

# Supplementary materials

# Supplementary table 1. Reason for not receiving RZV dose 2 (pooled TVC)

|  | **RZV** | | **Placebo** | |
| --- | --- | --- | --- | --- |
|  | **N=14,645** | | **N=14,660** | |
| **Reason** | **n** | **%** | **n** | **%** |
| Consent withdrawal, not due to an adverse event | 240 | 1.6 | 212 | 1.4 |
| Other | 204 | 1.4 | 156 | 1.1 |
| Non-serious adverse event | 155 | 1.1 | 57 | 0.4 |
| Serious adverse event and/or potential immune-mediated disease | 59 | 0.4 | 61 | 0.4 |
| Lost to follow-up | 39 | 0.3 | 44 | 0.3 |
| Migrated / moved from the study area | 16 | 0.1 | 8 | 0.1 |
| Protocol violation | 9 | 0.1 | 10 | 0.1 |
| Suspected herpes zoster episode | 8 | 0.1 | 33 | 0.2 |
| Total | 730 | 5.0 | 581 | 4.0 |

RZV, adjuvanted recombinant zoster vaccine; TVC, total vaccinated cohort, N, number of participants with at least one administered dose; n, number of participants in each category

# Figure S1. Plain language summary


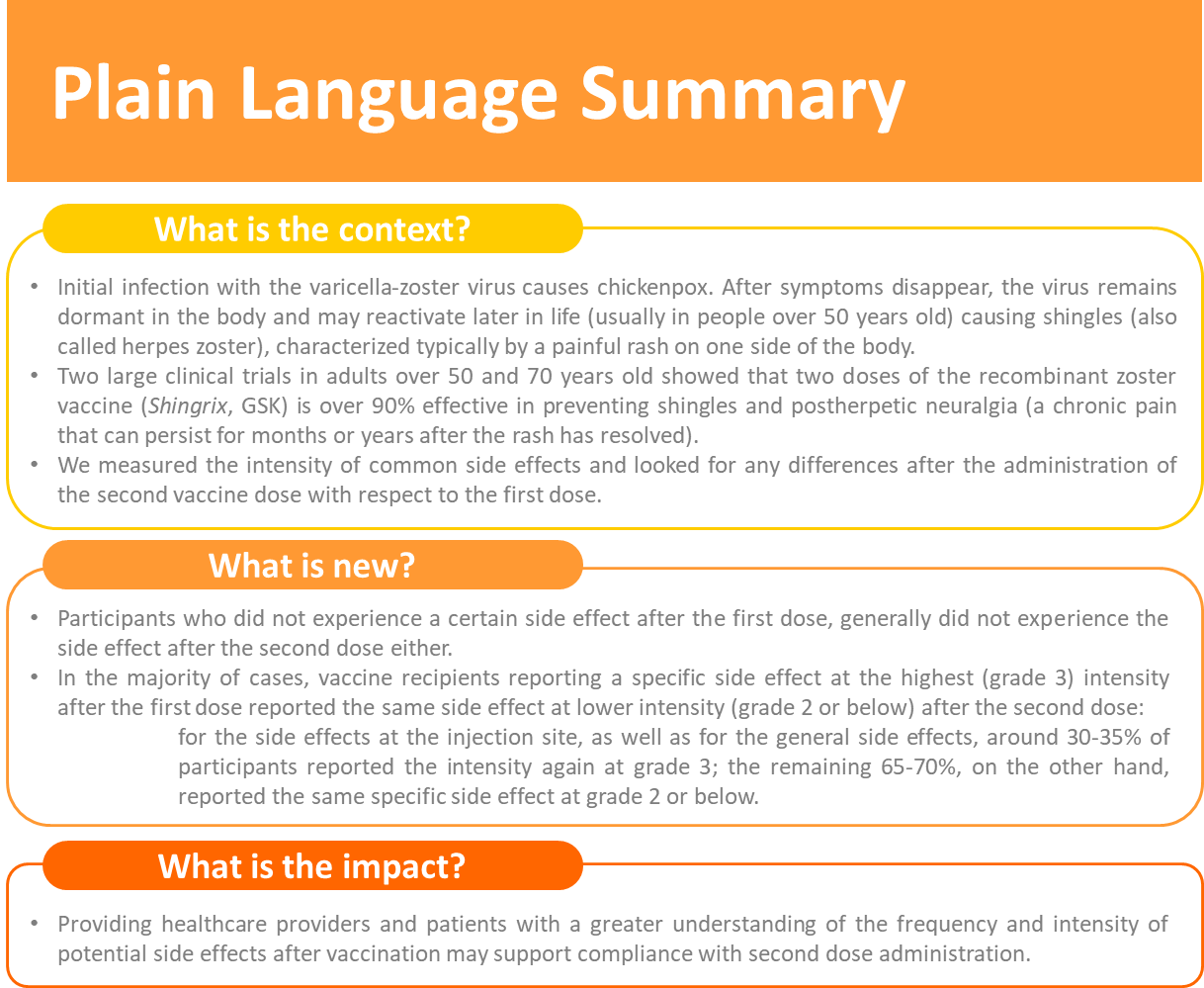

Supplement: Supplemental Material [file KHVI_A_1741312_SM9095.docx]
